# Contributions of Gut Bacteria and Diet to Drug Pharmacokinetics in the Treatment of Parkinson's Disease

**DOI:** 10.3389/fneur.2019.01087

**Published:** 2019-10-15

**Authors:** Sebastiaan P. van Kessel, Sahar El Aidy

**Affiliations:** Department of Molecular Immunology and Microbiology, Groningen Biomolecular Sciences and Biotechnology Institute, University of Groningen, Groningen, Netherlands

**Keywords:** levodopa, transporters, bioavailability, small intestinal bacterial overgrowth, gut motility

## Abstract

Parkinson's disease is the second-most common neurodegenerative disorder worldwide. Besides deciphering the mechanisms that underlie the etiology of the disease, it is important to elucidate the factors that influence the efficacy of the treatment therapeutics. Levodopa, which remains the golden treatment of the disease, is absorbed in the proximal small intestine. A reduction in levodopa absorption, leads to reduction in striatal dopamine levels and, in turn, an “off”-episode. In fact, motor fluctuations represent a major problem during the progression of the disease and alteration between “on” (mobility often with dyskinesia) and “off” (immobility, akinesia) episodes contribute to a decreased quality of life. Dietary amino acids can interfere with the absorption of levodopa from the gut lumen and its transport through the blood brain barrier. In addition, higher abundance of specific gut bacteria that restrict levodopa absorption plays a significant role in motor fluctuations in a subset of Parkinson's disease patients. Here, we review the impact of factors potentially interfering with levodopa absorption, focusing on levodopa transport, diet, and gut bacterial interference with the bioavailability of levodopa.

## Introduction

Parkinson's disease (PD) is the second-most common neurodegenerative disorder worldwide (1). In 2015–2016, 6.1–6.2 million individuals were diagnosed with PD all over the globe (1, 2). The prevalence of PD globally increases with age and peaks at 1.5% between 85 and 89 years of age (2). During the progression of PD, patients encounter increasing severity of symptoms, which is associated with rising costs for medical treatment, hospitalizations and nursing home care (3), besides a significant decrease in the quality of life (3–6). The aggregation of α-synuclein in Lewy bodies and loss of dopaminergic neurons (pars compacta) in the substantia nigra is the main feature observed in PD patients (7). Although the exact factors contributing to the etiology of PD are not well understood, the gut microbiota is likely to be a key contributor. This is evident from the alteration in gut microbiota composition detected in fecal samples of PD patients compared to healthy controls (HC) (8–12). Moreover, the production of short-chain fatty acids (SCFAs), the main metabolic products produced by the large intestinal bacteria, is reduced in PD patients (12). The latter has been shown to be involved in α-synuclein pathology in the gut in mouse models (13) supporting the hypothesis that α-synuclein pathology starts in the enteric nervous system (14), which synergizes with the finding of α-synuclein aggregates in colon tissue and appendix prior to the onset of PD (15, 16). Equally important to elucidating the mechanisms involved in the cause of PD is to uncover the microbial and dietary interference with the pharmacological treatment of the disease. Previous studies have shown that *Helicobacter pylori* (HP) can interfere with levodopa treatment and can bind to levodopa (3,4-dihydroxyphenylalanine; L-DOPA) (17, 18). Recently, we showed that bacteria can alter the levels of levodopa treatment in the gut (19) resulting in quenching the availability of the drug to be effective in the brain. This bacterial mediated reduction in levodopa absorbed from the small intestine would lead to reduction in striatal dopamine levels and an “off”-episode, especially in patients with advanced stage PD, who have a reduced capacity to store dopamine in the brain (20, 21). Besides, fluctuating levodopa plasma levels could result in increased pulsatile stimulation which is associated with dyskinesia (22). The pharmacological treatment of PD and the gastrointestinal (GI) dysfunction in PD have been extensively reviewed (23, 24), mainly from a clinical perspective. This review focuses on the impact of levodopa transport, gut bacterial degradation of PD medication, and its impact on drug bioavailability. Furthermore, we discuss the potential mediators that could lead to a vicious circle where certain conditions (i.e., proton pump inhibitors and gut motility) would favor the colonization of small-intestinal bacteria, ultimately restricting the absorption of levodopa.

## Administration Routes and Transport Process of Levodopa

The most common route for levodopa administration is orally via immediate-release or extended-release formulations of levodopa, where the latter might have potential benefits over other levodopa formulations, reviewed in Mittur et al. (25). Parenteral administration via subcutaneous injections are impossible due to the low solubility of levodopa (26) and continuous intravenous administration, although effective (27), is impractical, as it requires large volumes of daily injections. A promising alternative option to conventional levodopa therapy for advanced PD patients with motor fluctuations and dyskinesia is intestinal infusion of a levodopa/carbidopa gel via a nasoduodenal tube (28) or via gastrojejunostomy (22).

**Figure 1:**
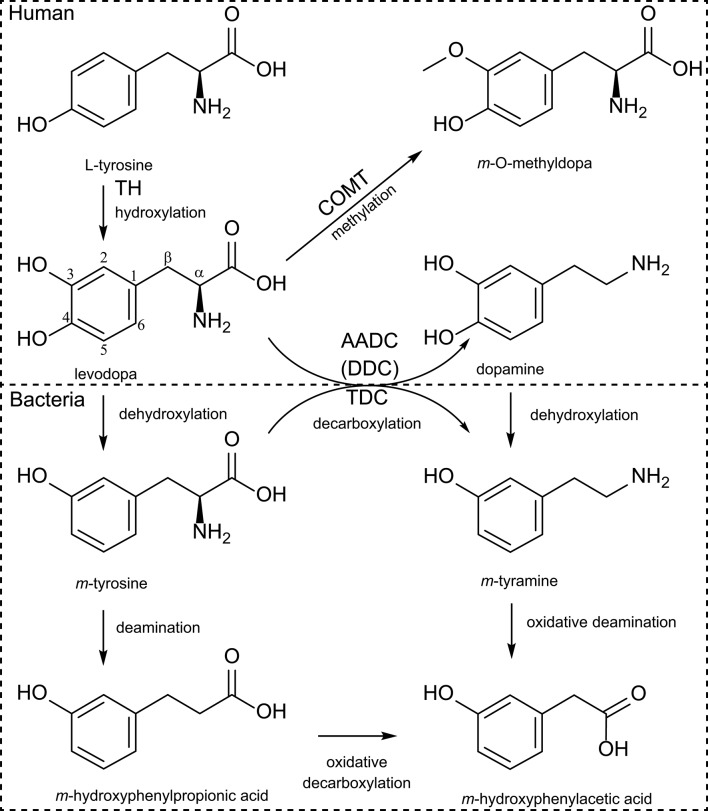
Human and bacterial levodopa metabolism. Levodopa is produced by hydroxylation of the meta-position of the phenyl-ring from tyrosine by TH (tyrosine hydroxylase) using molecular oxygen. Sequentially levodopa can be decarboxylated to the active neurotransmitter dopamine by the AADC [aromatic amino acid decarboxylase, also known as DDC (DOPA decarboxylase)], or can be methylated by COMT (catechol-O-methyltransferase). Bacterial TDC (tyrosine decarboxylase) can decarboxylate (*m*-)tyrosine to (*m*-)tyramine but also levodopa to dopamine. Furthermore, bacteria can dehydroxylate the para-hydroxyl group of either levodopa or dopamine and can sequentially deaminate the dehydroxylated products.

**Figure 2:**
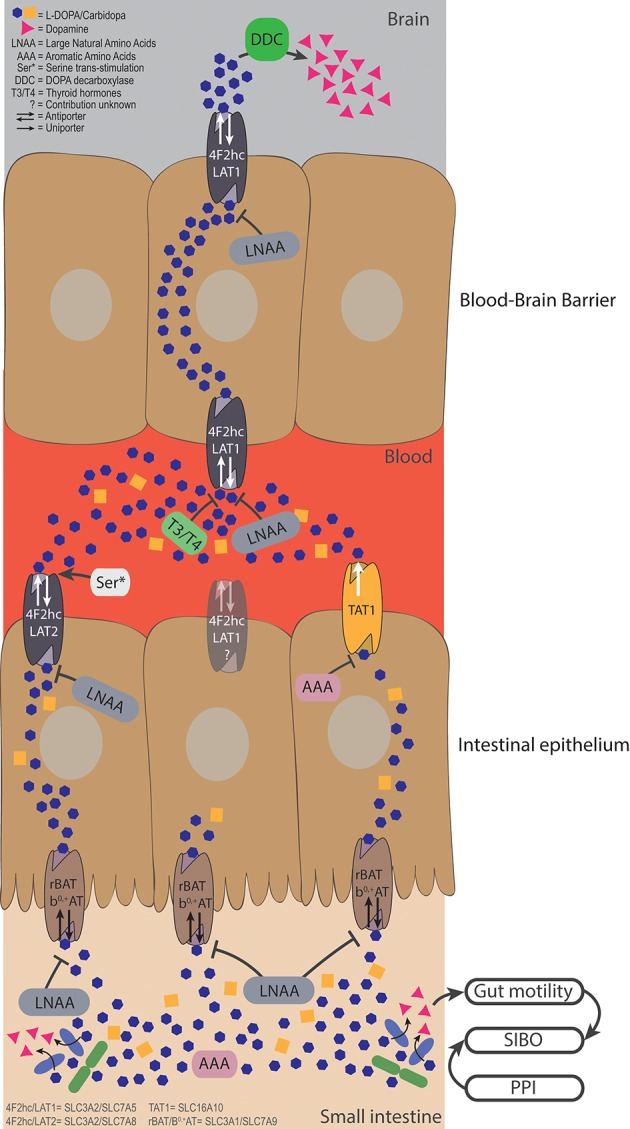
Bacterial degradation and dietary components restrict levodopa transport. Levodopa is taken up in the small intestine by the apical transporter rBAT/b^0,+^AT, and is sequentially is transported over the basolateral membrane by 4F2hc/LAT2 and TAT1. The uptake from the lumen can be compromised by LNAAs apically and by LNAAs and AAAs basolaterally. Bacterial degradation can interfere with levodopa before it is transported and elevate levels of dopamine in the lumen. Higher levels of luminal dopamine could affect the gut motility, which, in turn, could result in a state of small intestinal bacterial overgrowth, creating a vicious circle. The fraction of levodopa that ends up in the blood has to be transported over the BBB via 4F2hc/LAT1, which can be compromised by high levels of thyroid hormones (T3/T4), or LNAA. Serine left over from a late proteic meal, can trans-stimulate 4F2hc/LAT2 inducing higher efflux of levodopa in the circulation. Finally, the remaining levodopa will be converted to dopamine in the brain by DDC, to compensate the loss of striatal dopamine levels in PD patients.

When levodopa is administered orally, it is absorbed in the proximal small intestine (29), where it has to be actively transported from the lumen over the intestinal epithelial barrier into the blood stream (30). To prevent peripheral and intestinal levodopa metabolism by DOPA decarboxylase (DDC), peripheral DDC inhibitors, such as carbidopa, are co-administered with levodopa. Levodopa (Figure 1) is a non-proteinogenic large neutral amino acid (LNAA), and is therefore transported by amino acid transporters in the GI-tract and at the blood brain barrier (BBB) (Figure 2). The human body contains at least 11 different epithelial amino acid transport systems expressed in the intestine, 10 of which are also expressed in the renal epithelia, which was thoroughly reviewed before (31). Only two amino acid transporters are expressed on the blood brain barrier (BBB), LAT1 (SLC7A5) and SNAT5/11 (SLC38A5/11) (32). The amino acid transporters, which are most likely responsible for the transport of levodopa from the GI-tract to the blood and over the BBB, based on *in vitro/ex vivo* studies, are discussed below and summarized in Figure 2.

As a model for the BBB, a mouse brain endothelial cell line (MBEC4), was tested for the expression of 4F2hc/LAT1 (SLC3A2/SLC7A5) and [^3^H]-levodopa transport was evaluated in the presence of other amino acids (1:100 levodopa/amino acids). The study showed that tryptophan, tyrosine, phenylalanine, isoleucine, leucine, histidine, and 2-amino-2-norbornane-carboxylic acid (BCH), which is used as the defining synthetic amino acid for the L-system (consisting of LAT1 to 4) (33), inhibited at least 80% of the [^3^H]-levodopa uptake independent of Na^+^ (34). However, the potential contribution of 4F2hc/LAT2 (SLC3A2/SLC7A8) or other transporters were not addressed. Similar results were obtained in Caco2 cells (35–38), renal proximal tubular epithelial cells (39), and opossum kidney cells with either a high (HC) or a low (LC) Na^+^ influx. Comparing the HC and LC cell lines indicated that there was a minor contribution of Na^+^ dependent transport. The authors concluded that 4F2hc/LAT2 (apparent from BCH transport) and rBAT/b^0, +^ (SLC3A1/SLC7A9; apparent from the uptake of the rBAT defining amino acid dimer, cystine) were involved in levodopa transport (40). Although these studies indicate which transporters are involved in levodopa transport in the GI-tract, renal epithelia and the BBB, it remains unclear which specific transporter is involved.

Studies using *Xenopus laevis* oocytes, an ideal single-cell expression system for transporters due to its relatively large size and low background activity (41), showed that 4F2hc/LAT1 (from rat C6 glioma cells) (42), 4Fhc/LAT2 (43), rBAT/b^0, +^ (from rabbit intestine and human) (43, 44), and TAT1 (SLC16A10) (from rat intestine) (45) are independently responsible for levodopa transport. Only substrates with both positive and negative charges at the α-carbon (the relative positive and negative charges are from the amine-group and carboxyl-group from levodopa, respectively, Figure 1) are being able to be transported via 4F2hc/LAT1 (42). Importantly levodopa analogs (*m*-O-methylDOPA, α-methylphenylalanine, α-methyltyrosine, α-methylDOPA), gabapentin [γ-aminobutyric acid (GABA) analog], melphalan (a chemotherapeutic agent), and thyroid hormones (T3, triiodothyronine and T4, thyroxine) were able to inhibit transport of L-[^14^C]-phenylalanine, and thus levodopa (42), showing the broad range of potential levodopa transport inhibitors. In fact, anti-thyroid treatment in a 70-year-old male subject with PD on levodopa treatment had a beneficial effect on the exaggerated Parkinsonian tremor (46). The authors could not explain why the Parkinsonian tremor was aggravated by the presence of hyperthyroidism. However, a plausible explanation, which was not discussed, is the interference of exaggerated thyroid hormone levels with levodopa uptake in the brain. Thus, hyperthyroidism, which is prevalent at higher age, should be considered in PD patients (46).

In *X. laevis* oocytes expressing TAT1, around 80% of L-[^14^C]-tryptophan uptake was inhibited by tyrosine and tryptophan and about 40% was inhibited by phenylalanine, levodopa, and *m*-O-methylDOPA, indicating that TAT1 is an aromatic amino acid transporter partly responsible for levodopa uptake. Using N-acetylated amino acids, the authors concluded that the α-carboxyl group (Figure 1) is essential for substrate recognition by TAT1. Furthermore, it was shown that TAT1 is mainly expressed throughout in the rat GI-tract and in the liver, in particular, on the basolateral side of rat small intestine (45) (Figure 2). Using trans-well culturing and everted murine jejunal sacs, the authors concluded that 4F2hc/LAT2 (LAT1 was not tested) and TAT1 are responsible for the basolateral transport of levodopa (30). In contrast to 4F2hc/LAT1, 4F2hc/LAT2, and TAT1, which are expressed basolaterally, rBAT/b^0, +^AT is expressed apically and thus is mainly responsible for levodopa absorption from the intestinal lumen. Further characterization of rBAT/b^0, +^AT showed that the common co-administered inhibiters of peripheral levodopa degradation, carbidopa, benserazide (decarboxylase inhibitors) and entacapone [catechol-O-methyltransferase (COMT) inhibitor] were unable to compete with rBAT/b^0, +^AT mediated levodopa transport, indicating that other transporters/mechanisms are involved in the uptake of peripheral levodopa metabolism inhibitors (30). The transport of levodopa via other apical transporters, PAT1, SIT1/ACE2, ASCT2, and B^0^AT1/ACE2 (the main other natural amino acid transporter), expressed in *X. laevis* oocytes was investigated and showed that none of them was able to transport levodopa, indicating that rBAT/b^0, +^AT is the main apical levodopa transporter (30) (Figure 2).

## Effect of Diet and Age on the Bioavailability of Levodopa

Early studies *in vivo*, using radiolabeled levodopa ([^14^C]-levodopa) showed that ~90% of the total radioactivity is transported into the circulatory system as measured in urine samples after 48 h (47–49). Notably, only ~13% of the total radioactivity in blood plasma after the first hour was from intact levodopa, and decreased further overtime. When carbidopa was used in combination with levodopa the intact levodopa after the first hour increased to ~43% (47). These studies indicate that less than half of the administered levodopa would reach the brain and that approximately 10% of the total levodopa radioactivity is not absorbed and could end up in fecal samples. Moreover, levels of unabsorbed levodopa increase over age. For example, a 10-fold increase (24.6–35.4% vs. 2.7–3.5% recovered radioactivity) in levels of levodopa (including its metabolites) were detected in fecal samples of old rats (0.5–2 years old) when compared with their younger counterparts (5–15 weeks old) after oral administration of [^14^C]-levodopa (50). This was not related to an increased fecal excretion or decreased jejunal blood flow, suggesting that there is impaired uptake at older age (50). When levels of levodopa were measured over time in plasma (AUC), older animals (1–2 years) had a higher AUC and a longer half-life (T_1/2_) of systemic levodopa compared to younger animals (9–26 weeks), suggesting an age-dependent slower total body clearance of levodopa (50). Furthermore the study showed that the intestinal metabolism (mainly by DDC), which prevents levodopa to reach the brain and decreases over age, contributes the most to the increased systemic availability of levodopa at older age (50). The decreased clearance of levodopa at higher age in rats is in agreement with a study performed in healthy human subjects, who were administered levodopa without DDC inhibitors (51). Coherently, a higher AUC and systemic levodopa bioavailability (AUC_oral_/AUC_intravenous_) for levodopa was observed in elderly (71.0 years *n* = 9) compared to young subjects (21.8 years *n* = 8). Administration of carbidopa diminished the differences in systemic levodopa bioavailability between the two groups, while a higher AUC was still observed in the elderly group. This suggests a lower systemic clearance at higher age because carbidopa abolished the age differences in systemic levodopa bioavailability (51). In PD patients, age correlated significantly with higher levodopa (supplied with DDC inhibitor) AUC and decrease in clearance (52, 53). However, the high scatter in the correlation (*r*^2^ = 0.15–0.24) from that study implies that other factors besides age contribute to the variation among PD patients in the pharmacokinetics of levodopa (52).

Indeed, impaired uptake of [^14^C]-levodopa into the brain was observed when rats were supplied intravenously with the amino acids, phenylalanine, tryptophan, and to a lesser extent histidine (54). The same effects were reported in humans, for example, a clinical study showed that PD patients (*n* = 9), who received levodopa/carbidopa intravenously directly after a protein rich meal (containing LNAAs) or administration of LNAAs, had increased Parkinsonian symptoms. Similarly, when levodopa/carbidopa was taken orally, levodopa absorption from the intestine was delayed after a protein-rich meal (55). When levodopa/benzerazide (another DDC inhibitor) was infused intraduodenally, motor functions decreased after protein ingestion (56), indicating fluctuation in levodopa uptake in the brain. Nonetheless no decrease in levodopa absorption was observed (56) suggesting that the variability in plasma LNAAs, absorbed from the intestine, could be responsible for the fluctuating levodopa uptake in the brain (57). The authors concluded that during ingestion of regular (hospital) diets, 10% of the levodopa brain uptake variability is explained by LNAAs in plasma and the other 90% by levodopa plasma levels (57). These hospital diets contained 2–3.7-fold less LNAAs compared to other human studies [615 ± 105 μM (57) compared to 1,235–1,973 μM (55), 1,615–2,012 μM (58), 1,624–2,292 μM (56)] indicating that high LNAA levels do interfere with levodopa absorption in PD patients but are not solely responsible for the “on”–“off” fluctuations observed in PD patients. Notably, cationic (lysine) or small (glycine) amino acids had no effect on the “on”–“off” fluctuations (55). Using regional jejunal perfusion of levodopa in healthy human subjects it was shown that the LNAA L-leucine interfered with the levodopa absorption from small intestine (59), at least at high concentrations. This finding supports the involvement of the L-transport system for levodopa transport (as described above) from the intestine to the blood circulation, and, ultimately, to the brain (Figure 2).

*In vitro* data and clinical investigations on the effect of amino acids on the transport and bioavailability of levodopa clearly indicate that amino acids can interfere with the uptake of levodopa from the lumen or the systemic circulation. Therefore, low protein diets (LPD) or protein redistribution diets (PDR), where all dietary protein is ingested only during the evening meal, are proposed for PD patients with motor fluctuations (60). Refined physiologically based pharmacokinetic (PBPK) modeling for GI absorption (WB-ACAT, Whole Body—Advanced Compartmental Absorption and Transit Model) combined with dynamic flux balance analysis (which measures the flow of metabolites through a metabolic network) on an epithelial cell (sIEC) model for small intestine segmented into 7 parts (WB-ACAT-sIEC), was used to investigate the spatiotemporal relationship between amino acids and levodopa uptake kinetics (61). Simulation of levodopa absorption during an aproteic or proteic meal showed that that dietary intervention would be beneficial for PD patients with Hoehn and Yahr scale 3/4 (HY3/4; HY describes the disease progression from (mild = 1) to severe = 5) (61). These findings are in agreement with the guidelines for PD treatment, where dietary interventions are proposed for advanced PD patients (20, 21). Comparing a LPD (*in silico* administration of 0.8 g/kg amino acids together with 200 mg levodopa) vs. a PRD (assuming a high fraction of amino acids present in the systemic circulation before the morning levodopa dose) in the WB-ACAT-sIEC model showed a cumulative increase in AUC of levodopa during PRD. Furthermore, the AUC after a morning levodopa dose was higher (11.23%) during PRD than during a fasting state, which was attributed to a higher influx of residual systemic LNAA from the last protein meal taken the evening before levodopa administration. This higher influx through the basolateral antiporter induced a higher efflux of levodopa (trans-stimulation) into the circulation (61) (Figure 2). Although PRD could provide short-term benefits as evident by the reported response rates of >80% (60), it might not provide a long-term solution as it is undesired by patients and is an imbalanced diet (20, 21) that results in weight loss among patients (60). Extending the WB-ACAT-sIEC model with kidney and brain compartments and setting the objective function (a desired outcome) for optimizing levodopa transport across the BBB revealed that threonine, serine and asparagine resulted in the highest brain bioavailability of levodopa. This led the authors to propose that a serine-rich meal taken after the last levodopa treatment could be beneficial for the levodopa bioavailability (61). Nonetheless, sensitivity analyses (i.e., the variable that contributes most to the dependent outcome) showed that intestinal loss of levodopa was the most influential factor on levodopa bioavailability (61). Indeed, changes in the levels of levodopa in the small intestine are affected by gut bacterial interference (17, 19), as discussed in the next section.

## Gut Bacterial Interference With Levodopa Bioavailability

Levodopa is a non-proteinogenic amino acid produced by the hydroxylation at the meta-position of the phenyl ring of tyrosine. Subsequently, levodopa can be converted to dopamine by DDC or to *m*-O-methylDOPA by COMT methylating of the *m*-hydroxyl group in the human body (Figure 1). The microbiota also poses enzymes able to perform similar or additional reactions, which metabolize levodopa. In the early 70s, a study, comparing the metabolic profile of germ-free and conventional rats, showed production of *m*-hydroxyphenylacetic acid and *m*-hydroxyphenylpropionic acid (Figure 1) only in conventional rats when fed with levodopa, suggesting that a bacterial dehydroxylation reaction was involved (62). When rat caecal content was incubated with levodopa or dopamine for 6 days also *m*-tyramine was found, confirming earlier findings in humans (63). Metabolites were detected over periods of 3 days in the urine indicating that the detected metabolites could originate from in the large intestine, which is supported by the caecal incubations (62). Since the main site of levodopa absorption is the proximal small intestine, it is unlikely that bacterial metabolism of levodopa in the large intestine would affect the drug bioavailability. Therefore, it is crucial to investigate potential bacterial interference with levodopa treatment in the proximal small intestine.

Recently, we showed that gut bacteria harboring tyrosine decarboxylases (TDC), mainly enterococci, can effectively decarboxylate levodopa to dopamine in the small intestine of rat. The study concluded that the natural variation of the *tdc*-gene negatively correlated with the levodopa levels in the blood of rats and positively correlated with the daily dose requirement of levodopa in PD patients (19). High abundance of these bacteria in PD patients, which could be caused by small intestinal overgrowth (SIBO), could have implications on the absorption of levodopa from the small intestine (Figure 2). To assess the contribution of those bacteria to the bioavailability of levodopa in PD patients, we are currently performing further clinical studies.

In healthy conditions, SIBO is prevented by the ileocecal valve, pancreatic enzyme activity, gut motility and gastric acid (64). Importantly in PD patients, the prevalence of gut motility dysfunction (constipation) and proton pump inhibitor (PPI) usage is relatively high (77.1 and 39.6% respectively, *n* = 39) (65) and is associated with SIBO (66). In patients (*n* = 200) with gastroesophageal reflux disease using PPIs, varying from 2 months to 7 years, SIBO was detected in 50% of the cases and was significantly higher than in healthy controls (*n* = 50) (66). Studies looking at the alteration of the microbiota in subjects using PPIs showed increased levels of Bacilli (including *Lactobacillus, Staphylococcus*, and *Enterococcus*) in fecal samples (67, 68). In duodenal samples, SIBO was also observed in 56% of patients on PPIs (*n* = 25) and included mainly genera from the Bacilli class (69). Bacterial species from the Bacilli class are of importance as they harbor TDCs, which are able to interfere with levodopa levels (19). When SIBO is eradicated in PD patients with *Helicobacter pylori* infection using rifaximin, a common non-absorbable antibiotic used to treat SIBO (70), motor fluctuations were improved as apparent from the significant decreased delayed “on” episodes/day and daily “off” time, although no significant increase in levodopa pharmacokinetics was observed (71). The underlying explanation of improved motor fluctuations following SIBO eradication remains to be elucidated. However, a plausible explanation is that eradication of bacterial degradation of levodopa in the small intestine altered levels of the levodopa metabolite, dopamine, in the small intestinal lumen (19), and/or eliminated SIBO-induced small intestinal inflammation (71).

In 2001, investigators observed a clinical improvement in PD patients after treatment with antibiotics used to eradicate *Helicobacter pylori* in two almost identical reports. When HP-infections were treated, the mean AUC of levodopa in the blood significantly increased by ~1.2-fold. A UPDRS-III motor examination showed indeed a significant decrease in motor score (72, 73). A follow-up study confirmed these findings in a larger cohort (*n* = 17) and showed that either 2 weeks or 3 months after HP eradication, PD patients had higher levodopa blood levels (AUC) and lower UPDRS-III motor scores compared to before the eradication (18). Other studies did not find a significant difference in pharmacokinetics (74) or LEDD (levodopa equivalent daily dose) (75, 76) of levodopa between PD patients tested positive or negative for HP infection. In addition, no motor improvement (UPDSR-III) was found after HP eradication in 34 patients (75). Despite the discrepancy among studies, *Helicobacter pylori* might still play a significant role in drug absorption. The mechanism of *Helicobacter pylori* affecting the levodopa absorption is unclear, one possible explanation for altered drug absorption might be the gastric acidity, which is altered by *Helicobacter pylori* infection and therefore interferes with drug pharmacokinetics of levodopa, delavirdine, and thyroxine (77). Interestingly, an *in vitro* study showed that adhesins exposed on the outer membrane of *Helicobacter pylori* might bind to levodopa and therefore might contribute to the lower pharmacokinetics in *Helicobacter pylori* infected PD patients (17). No follow-up studies were published and it remains to be elucidated which adhesin(s) are responsible for binding levodopa. Besides, whether the antibiotic cocktail used to treat *Helicobacter pylori* infections (1,000/500 mg amoxicillin/clarithromycin) could also eradicate other bacterial species in the small intestine, which might interfere with the availability of levodopa, and thus could be the actual reason behind the observed increase in blood levels of levodopa, was not investigated.

## Effect of Dopamine and Dopamine Agonists on Gut Motility

Bacterial species from the Bacilli class, especially enterococci, are able to produce luminal dopamine (19). Importantly, dopamine and their agonists have been shown to affect the gut motility (discussed below), which could potentially favor the colonization of levodopa decarboxylating bacteria (19) (Figure 2). In addition, the dopamine agonists, which are usually used in combination with levodopa treatment, could have a similar effect on influencing gut motility to favor colonization of specific bacterial species. Therefore, studies investigating the effects of dopamine on gut motility of rodents, dogs, and humans were reviewed, with a complete overview in Table 1.

**Table 1:** Studies investigating the effects of dopamine and dopamine agonists on gut motility in rodents, dogs and humans.

**Study**	**Organism**	**Method**	**Tissue**	**Effect on motility**	**Tested agonists (μM)**	**Dopamine receptor antagonist (μM)**	**Adrenergic receptor antagonist (μM)**	**Other inhibitors**	**Effect inhibited by**	**Conclusion**
Zar et al. (78)	Guinea pig	Organ bath	Ileum; longitudinal muscle; electrical field stimulation	Relaxation	Dopamine (1–100), Bromocriptine (0.15–15)	Pimozide (1)	Phentolamine (5), Metoclopramide (90)	None	Phentolamine (only DA)	Inhibition of longitudinal muscle motility through α-adrenergic receptors
Görich et al. (79)	Guinea pig	Organ bath	Ileum; longitudinal fixation (reserpine pretreatment)	Inhibitory	Dopamine, Noradrenaline, Clonidine (and tyramine) (1–100)	Metoclopramide (1–30), sulpiride (1–300), domperidone (0.01–1), pimozide (0.01–0.1) cis-flupentixol (0.1–1)	Tolazoline (0.3–3)	Reserpine (VMAT2 inhibitor)	Metoclopramide, sulpiride, tolazoline	Inhibition of motility by all compounds tested. Potentially through α-adrenergic receptors. The potency (pA2^*^) of metoclopramide and sulpiride was not different between dopamine or norepinephrine, indicating an α-adrenergic inhibition, confirmed by tolazoline
Lucchelli et al. (80)	Guinea pig	Organ bath	Jejunum; longitudinal fixation; methacholine induced contraction	Relaxation	Dopamine (1–000), Apomorphine (3–100), Bromocriptine (1–56), Fenoldopam (1,000), [and tyramine 1–3,000 (data not shown)]	Haloperidol (1,3), *cis*-flupenhixol (1), SCH-23390 (1,3)	Phentolamine (1,3), propranolol (0.3,1,3,10)	Reserpine (I.P. 5 mg/kg), TTX (0.3)	Phentolamine (only ~7%) and propranolol (up to ~45%)	Relaxation of tissue of all tested compounds (Reserpine, had no effect on DA induced relaxation, and a minor effect on the others). Slight inhibition of phentolamine (α-adrenoceptor antagonist) and propranolol (ß-adrenoceptor antagonist). Inconclusive which receptor is involved
Kirschstein et al. (81)	Rat	Organ bath	Duodenum, jejunum, ileum; longitudinal fixation	Relaxation and Constriction	Dopamine (100)	SCH-23390 (1), raclopride (1)	Propranolol (3), Prazosin (30)	None	All tested	Contraction and relaxation observed in duodenum and jejunum, relaxation only observed in Ileum. Contraction inhibition by SCH-23390 and raclopride, relaxation inhibition by propranolol and prazosin
Zhang et al. (82)	Rat	Organ bath	Distal colon; longitudinal strips	Inhibitory	Dopamine (3–30)	SCH-23390 (10), Supiride (10)	Not tested	TTX (1)	SCH-23390	Dopamine inhibited the spontaneous contractions with EC50 = 8.3μM and was not affected by TTX. The inhibitory affect was affected only by D1R antagonist SCH-23390
Zizzo et al. (83)	Mouse	Organ bath	Ileum; longitudinal fixation	Inhibitory	Dopamine (1–300), SKF-38393 (0.003–100)	SCH-23390 (3,10), Sulpiride (10), Domperidone (5)	Propranolol (10) SR-59230A (0.1), Phentolamine, (10) Yohimbine (10)	DDA (10), Apamin (0.1), Charybdotoxin (0.1), Iberiotoxin (0,1), TTX (1), L-NAME (100), Atropine(1), DPCPX (10), DMPX (10), MRS-1220 (0.1), Methysergide (1)	SR-59230, Phentolamine, Yohimbine (at high concentration of DA), SCH-23390 and SCH-23390 in combination with Sulpiride or Domperidone	Contractibility was inhibited by dopamine and SKF-38933 (D1R agonist), at high concentrations adrenoceptor antagonists (SR-59230, phentolamine, yohimbine) slightly prevented the inhibitory effect of dopamine. D2 antagonists sulpiride and domperidone had little effect on the inhibitory effect of dopamine, except when combined with SCH-23390 (D1R antagonist) which induced a stronger effect then SCH-23390 alone. Suggesting a synergic contribution of D1 and D2 receptors
Auteri et al. (84)	Mouse	Organ bath	Colon; circular muscle strips; Carbachol precontracted or electrical field stimulation	Relaxation/Inhibitory	Dopamine (1–300), SKF-38393 (up to 100), bromocriptine (0.3–100), isoproterenol	SCH-23390 (3), domperidone (5)	Prazosin (1), Yohimbine (1), propranolol (1), SR-59230A (0.1)	TTX (1), ω-conotoxin (0.1), SNX-482 (0.1), ω-agatoxin TK (0.1), L-NAME (100) MRS-2179 (1),	Domperidone (during carbochol contraction); SCH-23390 (during electrical field stimulation)	Relaxation induced by DA via a D2-like receptors; Not dependent on NO or P2Y1 receptors; Not affected by adrenergic antagonists; not dependent on enteric neuronal action potential or on modulation of neurotransmitter release; SCH-23390 increased basal tone and the amplitude of the spontaneous contractions; Relaxation of bromocriptine is inhibited by domperidone
Walker et al. (85)	Mouse	Organ bath	Distal colon (WT and DAT-/-); Longitudinal fixation; Electrical field stimulation	Inhibitory	Dopamine (0.01–300)	SCH-23390 (10), sulpiride (10)	Not tested	None	SCH-23390/sulpiride	Dopamine was only tested on WT distal colon and showed a inhibitory effect (EC50 = 4.5 μM), which was slightly abolished by SCH-23390/sulpiride mixture (EC50 = 12.9 μM, single applications of antagonist were not performed)
Fioramonti et al. (86)	Dog	Implanted Ni/Cr electrodes	Duodenum and jejunum	Inhibitory	Intracerebroven-tricularly dopamine (10 ug/kg); Intravenous dopamine (100 μg/kg)	None	None	None	NA	Decreased the duration of the migrating motor complex episodes in the small intestine 1 h before a meal compared to controls (from 9.4 to 3.4 h and 7.8 to 2.4 h in duodenum and jejunum), although intravenously (100 μg/kg) this effect was not observed
Bueno et al. (87)	Dog	Implanted strain gauge transducers	Ascending, traverse, descending colon	Inhibitory and Inducing	Iv injections of dopamine at 1 mg/kg/h or bromocriptine 40 ug/kg/h	Haloperidol (0.2 mg/kg)	Phentolamine (0.1 mg/kg), Tolazoline (2 mg/kg), Prazosin (0.2 mg/kg), propranolol (0.5 mg/kg)	None	Phentolamine, prazosin and haloperidol for dopamine inhibitory effect,	Dopamine had a inhibitory effect on the ascending and transverse colon and a inducing effect on the descending colon MMCs. Bromocriptine had a inducing effect in the whole colon MMCs; Potentially through adrenergic and dopaminergic action
Marzio et al. (88)	Human, healthy	Intestinal radiopaque tube consisting of four polyvinyl catheters with 4 side openings equally spread perfused with 1.59 ml/min with distilled water. Closure of the openings gives rise 100 mm hg/sec	Duodenum, proximal jejunum	Inducing	Intravenously dopamine 5 μg/kg/min for 15 min	Domperidon (10 mg) and sulpiride (100 mg)	None	None	Domperidon and sulpiride	Dopamine induced phase-III like MMCs in the duodenum, similar to spontaneous phase-III MMCs, although a slight longer period of complete inhibition after phase-III MMCs; Domperidon and sulpiride prevented the inducing phase-III MMCs effect
Marzio et al. (89)	Human, healthy	Nasoduodenal probe consisting of 5 polyethylene catheters with evenly spaced openings 20 cm apart continuously perfused with 0.5 ml/min distilled water	Stomach, Duodenum, Proximal Jejunum	Inducing/Inhibitory	Intravenously dopamine 5 μg/kg/min for 15 min	Domperidon (20 mg)	None	None	Domperidon	Dopamine induced phase-III like MMCs during fed state in the small intestine, which was inhibited by domperidone, and decreased the motility of the stomach. After the phase-III MMCs a short period of complete quiescence was observed
Levein et al. (90)	Human, healthy	Paracetamol AUC; orocaecal transit time	Mouth -> Ileum	Inhibitory	Intravenously dopamine 5 μg/kg/min	None	None	None	NA	Dopamine reduced the AUC(60 min) of paracetamol significantly, associated with a delayed gastric emptying; OCT time was significantly longer then controls indicating a delayed gastric emptying and gut motility
Dive et al. (91)	Human, critically ill adults under mechanical ventilation without suffering from active gastro-intestinal disease	Multilumen tube consisting of polyvinyl catheters with side openings, 1.5 cm apart for stomach and 10 cm apart for duodenum continuously perfused with 0.2 ml/min distilled water	Stomach, duodenum	Inhibitory/Inducing	Intravenously dopamine 4 μg/kg/min	None	None	None	NA	Decreased number of contractions in the gastric antrum (only significant during fasting) and induced phase III motor activity in the duodenum (only significant during feeding)

* *pA2, the concentration that produces a 2-fold shift in the agonist concentration-response curve; **Dopaminergic antagonists:** SCH-23390, D1 receptor antagonist; Domperidone, Haloperidol, Metoclopramide, Pimozide, Raclopride, Sulpiride, D2 receptor antagonist; cis-flupentixol, D1 and D2 receptor antagonist; **Adrenergic antagonists**: Tolazoline, Phentolamine, Prazosin, α1 adrenergic receptor antagonist; Yohimbine, α2 adrenergic receptor antagonist; Propranolol, ß adrenergic receptor antagonist; SR-59230A, β3-adrenoceptor antagonist; **Other antagonists and inhibitors**: Apamin, SK_Ca_ channel blocker; Atropine, Muscarinic receptor blocker; Carbachol, Cholinergic agonist; Charybdotoxin, IK_Ca_-Bk_Ca_ channel blocker; DDA, Adenylyl cyclase inhibitor; DMPX, Adenosine A2 receptor antagonist; DPCPX, Adenosine A1 receptor antagonist; Iberiotoxin, BK_Ca_ channel blocker; L-NAME, NO synthase inhibitor; Methysergide, 5-HT receptor antagonist; MRS-1220, Adenosine A3 receptor antagonist; MRS-2179, Purinergic P2Y1 receptor antagonist; Reserpine, VMAT inhibitor; SNX-482, P/Q-type Ca^2+^ channel blocker; TTX, Na^+^voltage-gated neural channel blocker; ω-agatoxin TK, R-type Ca^2+^ channel blocker; ω-conotoxin, N-type Ca^2+^ channel blocker*.

Using electrical field stimulation (EFS) on longitudinal muscle strips of guinea pig ileum in organ baths, dopamine (1–100 μM) and bromocriptine (0.15–15 μM), a dopamine agonist used in PD treatment, inhibited the cholinergic twitch up to ~46 and ~82%, respectively. Neither dopamine antagonists, metoclopramide nor pimozide prevented the observed inhibition by dopamine or bromocriptine. When using the α-adrenoceptor antagonist, phentolamine, only the observed inhibition of dopamine but not of bromocriptine was rescued, indicating that dopamine acts through the α-adrenoceptors (78). The same conclusions on the inhibitory effect of dopamine were shown in an almost identical study using ileum of guinea pig (79). Notably, tyramine, a product of bacterial TDC, resulted in similar inhibitions of cholinergic twitch (79). Dopamine, bromocriptine, and to a lesser extent tyramine, were also able to relax methacholine-contracted jejunal tissues from guinea pig (80). In rats, dopamine initiated directly a short longitudinal contraction followed by relaxation within 5 min in the duodenum and jejunum. However, in the ileum, only relaxations were observed (81). In addition, dopamine had also an inhibitory effect on the spontaneous contractions of longitudinal muscle strips from rat distal colon (82). The motility of mouse longitudinal fixed ileum (83), circular muscle strips of colon (84) and longitudinal fixed colon (85) were all inhibited by dopamine and in the latter study also by bromocriptine, attributed to dopaminergic and/or adrenergic receptors. In dogs, the gut motility of the small intestine (86) and the colon (87) was monitored *in vivo* using implanted electrodes. Injection of dopamine (10 μg/kg) intracerebroventricularly 1 h before a meal decreased the duration of the migrating motor complex (MMC; intestinal motility pattern of the interdigestive state) episodes in the small intestine compared to controls, although this effect was not observed when dopamine was injected intravenously (100 μg/kg) (86). In the colon, a similar inhibition was observed, although with a 10 times higher concentration of dopamine (1 mg/kg/h) injected intravenously (87). Importantly, bromocriptine had an opposite effect, where it induced the colon motility instead (87). In fasted human subjects, intravenous administration of dopamine (75 μg/kg in 15 min) induced phase-III like MMCs (last phase in the MMC cycle which consists of strong contractions to completely occlude the lumen) in the duodenum (88), which is in contrast to the previous studies in rodents (organ bath experiments) and dogs. The MMCs were similar to spontaneous phase-III MMCs, although with a slight longer period of complete inhibition after phase-III MMCs (88). Similar results were found in terminally ill patients (91). A follow up study in humans during fed state showed that dopamine disrupted the fed state MMCs and induced phase-III like MMCs, followed by a short period of complete quiescence (phase-I like MMCs), which was inhibited by the dopamine receptor D2 blocker (DRD2) domperidone, suggesting the involvement of peripheral D2 receptors (89). Lastly, when the gut motility was investigated using orocaecal transit time (OCT) and paracetamol pharmacokinetics as gastric emptying marker during intravenous injection of dopamine (90), a reduction in the AUC_t = 60min_ of paracetamol was observed. This suggests that dopamine causes delayed OCT time, which could be due to delayed gastric emptying and a decrease in gut motility (90). Functional studies investigating the dopamine receptors in the GI-tract of mouse showed that the dopamine receptor D2 (Drd2) is important for gut motility. Mice lacking Drd2, but not Drd3, receptor showed an increased gut transit time compared to the controls (92) suggesting that endogenous dopamine has an inhibitory effect on intestinal motility (92). The findings confirm the earlier organ bath experiments with rodent tissue. In summary, these studies (Table 1) show that in rodents and dogs the GI motility is inhibited by dopamine through dopaminergic and adrenergic receptors.

In contrast, in humans, dopamine seems to inhibit stomach motility and induce phase-III like MMCs followed by a short time of quiescence through dopaminergic receptors. A potential explanation of the discrepancy among the human and the animal studies might be the experimental setup. In rodents, dissected intestinal parts were placed in an organ bath *ex vivo* and in dogs electrodes were implanted on the basal side of segments of the GI-tract (86, 87). In contrast, in human studies, nasojejunal luminal-tubes consisting of catheters with side openings were fluoroscopically placed in the GI-tract and perfused with 0.2–1.59 mL/min water (88, 89, 91). The latter might induce an altered gut motility *per se* in a non-physiological manner. More studies should be conducted to test the effects of dopamine on the gut motility in humans, and especially in PD patients, who might already have an altered gut motility (4).

## Conclusions and Future Perspectives

The “on”/“off” motor fluctuations in PD patients are highly dependent on the pharmacological treatment and factors contributing to its efficacy. Dietary amino acids and gut bacterial interference with levodopa treatment can contribute to the reduction of levodopa dosage absorbed in the small intestine, thereby restrict the effectiveness of the treatment. Especially luminal dopamine, which is produced by gut bacterial degradation of levodopa and is affecting the gut motility, would enhance the overgrowth of these bacteria in the small intestine and result in a vicious circle that enhances SIBO. The effect of dopamine on (small) intestinal motility, urges the investigation of the effect luminal dopamine and dopamine agonists on the gut motility of PD patients. Finally, it is crucial to accurately measure levels of SIBO in PD patients, especially in those who administer PPIs, and to diagnose other possible underlying diseases, such as hyperthyroidism. These precautions will help reduce the factors contributing to compromised levodopa bioavailability and the unwarranted side effects that result from increased frequency of dosage treatment regimen.

## Author Contributions

SK wrote the original manuscript that was reviewed and edited by SE. Funding was acquired by SE.

### Conflict of Interest

The authors declare that the research was conducted in the absence of any commercial or financial relationships that could be construed as a potential conflict of interest.

## References

[1] Feigin VL Abajobir AA Abate KH Abd-Allah F Abdulle AM Abera SF Global, regional, and national burden of neurological disorders during 1990–2015: a systematic analysis for the Global Burden of Disease Study 2015. Lancet Neurol. (2017) 16:877–97. 10.1016/S1474-4422(17)30299-5 28931491 PMC5641502

[2] Dorsey ER Elbaz A Nichols E Abd-Allah F Abdelalim A Adsuar JC Global, regional, and national burden of Parkinson's disease, 1990–2016: a systematic analysis for the Global Burden of Disease Study 2016. Lancet Neurol. (2018) 17:939–53. 10.1016/S1474-4422(18)30295-3 30287051 PMC6191528

[3] Kowal SL Dall TM Chakrabarti R Storm MV Jain A. The current and projected economic burden of Parkinson's disease in the United States. Mov Disord. (2013) 28:311–8. 10.1002/mds.25292 23436720

[4] Barone P Antonini A Colosimo C Marconi R Morgante L Avarello TP. The PRIAMO study: a multicenter assessment of nonmotor symptoms and their impact on quality of life in Parkinson's disease. Mov Disord. (2009) 24:1641–9. 10.1002/mds.22643 19514014

[5] Chapuis S Ouchchane L Metz O Gerbaud L Durif F. Impact of the motor complications of Parkinson's disease on the quality of life. Mov Disord. (2005) 20:224–30. 10.1002/mds.20279 15384126

[6] Martinez-Martin P Rodriguez-Blazquez C Kurtis MM Chaudhuri KR. The impact of non-motor symptoms on health-related quality of life of patients with Parkinson's disease. Mov Disord. (2011) 26:399–406. 10.1002/mds.23462 21264941

[7] Braak H Tredici K Del Rüb U de Vos RA Jansen Steur EN Braak E. Staging of brain pathology related to sporadic Parkinson's disease. Neurobiol Aging. (2003) 24:197–211. 10.1016/S0197-4580(02)00065-9 12498954

[8] Hill-Burns EM Debelius JW Morton JT Wissemann WT Lewis MR Wallen ZD. Parkinson's disease and Parkinson's disease medications have distinct signatures of the gut microbiome. Mov Disord. (2017) 32:739–49. 10.1002/mds.26942 28195358 PMC5469442

[9] Keshavarzian A Green SJ Engen PA Voigt RM Naqib A Forsyth CB. Colonic bacterial composition in Parkinson's disease. Mov Disord. (2015) 30:1351–60. 10.1002/mds.26307 26179554

[10] Qian Y Yang X Xu S Wu C Song Y Qin N. Alteration of the fecal microbiota in Chinese patients with Parkinson's disease. Brain Behav Immun. (2018) 70:194–202. 10.1016/j.bbi.2018.02.016 29501802

[11] Scheperjans F Aho V Pereira PAB Koskinen K Paulin L Pekkonen E. Gut microbiota are related to Parkinson's disease and clinical phenotype. Mov Disord. (2015) 30:350–8. 10.1002/mds.26069 25476529

[12] Unger MM Spiegel J Dillmann K Grundmann D Philippeit H Bürmann J. Short chain fatty acids and gut microbiota differ between patients with Parkinson's disease and age-matched controls. Parkinsonism Relat Disord. (2016) 32:66–72. 10.1016/j.parkreldis.2016.08.019 27591074

[13] Sampson TR Debelius JW Thron T Janssen S Shastri GG Ilhan ZE. Gut microbiota regulate motor deficits and neuroinflammation in a model of Parkinson's disease. Cell. (2016) 167:1469–80.e12. 10.1016/j.cell.2016.11.018 27912057 PMC5718049

[14] Hawkes CH Del Tredici K Braak H. Parkinson's disease: a dual-hit hypothesis. Neuropathol Appl Neurobiol. (2007) 33:599–614. 10.1111/j.1365-2990.2007.00874.x 17961138 PMC7194308

[15] Killinger BA Madaj Z Sikora JW Rey N Haas AJ Vepa Y. The vermiform appendix impacts the risk of developing Parkinson's disease. Sci Transl Med. (2018) 10:eaar5280. 10.1126/scitranslmed.aar5280 30381408 PMC6319259

[16] Shannon KM Keshavarzian A Dodiya HB Jakate S Kordower JH. Is alpha-synuclein in the colon a biomarker for premotor Parkinson's Disease? Evidence from 3 cases. Mov Disord. (2012) 27:716–9. 10.1002/mds.25020 22550057

[17] Niehues M Hensel A. *In-vitro* interaction of L-dopa with bacterial adhesins of Helicobacter pylori: an explanation for clinicial differences in bioavailability? J Pharm Pharmacol. (2009) 61:1303–7. 10.1211/jpp/61.10.0005 19814861

[18] Pierantozzi M Pietroiusti A Brusa L Galati S Stefani A Lunardi G. Helicobacter pylori eradication and l-dopa absorption in patients with PD and motor fluctuations. Neurology. (2006) 66:1824–9. 10.1212/01.wnl.0000221672.01272.ba 16801644

[19] van Kessel SP Frye AK El-Gendy AO Castejon M Keshavarzian A van Dijk G. Gut bacterial tyrosine decarboxylases restrict levels of levodopa in the treatment of Parkinson's disease. Nat Commun. (2019) 10:310. 10.1038/s41467-019-08294-y 30659181 PMC6338741

[20] Olanow CW Koller WC. An algorithm (decision tree) for the management of Parkinson's disease: treatment guidelines. Am Acad Neurol Neurol. (1998) 50:S1–57. 10.1212/WNL.50.3_Suppl_3.S1 9524552

[21] Olanow CW Watts RL Koller WC. An algorithm (decision tree) for the management of Parkinson's disease (2001): treatment guidelines. Neurology. (2001) 56:S1–88. 10.1212/WNL.56.suppl_5.S1 11402154

[22] Olanow CW Kieburtz K Odin P Espay AJ Standaert DG Fernandez HH. Continuous intrajejunal infusion of levodopa-carbidopa intestinal gel for patients with advanced Parkinson's disease: a randomised, controlled, double-blind, double-dummy study. Lancet Neurol. (2014) 13:141–9. 10.1016/S1474-4422(13)70293-X 24361112 PMC4643396

[23] Connolly BS Lang AE. Pharmacological treatment of Parkinson disease: a review. JAMA. (2014) 311:1670–83. 10.1001/jama.2014.3654 24756517

[24] Fasano A Visanji NP Liu LWC Lang AE Pfeiffer RF. Gastrointestinal dysfunction in Parkinson's disease. Lancet Neurol. (2015) 14:625–39. 10.1016/S1474-4422(15)00007-1 25987282

[25] Mittur A Gupta S Modi NB. Pharmacokinetics of Rytary®, an extended-release capsule formulation of carbidopa–levodopa. Clin Pharmacokinet. (2017) 56:999–1014. 10.1007/s40262-017-0511-y 28236251 PMC5563351

[26] Lundqvist C. Continuous levodopa for advanced Parkinson's disease. Neuropsychiatr Dis Treat. (2007) 3:335–48. 19300565 PMC2654791

[27] Hardie RJ Lees AJ Stern GM. On-off fluctuations in Parkinson's disease. A clinical and neuropharmacological study. Brain. (1984) 107 (Pt 2):487–506. 10.1093/brain/107.2.487 6722513

[28] Nyholm D Nilsson Remahl AIM Dizdar N Constantinescu R Holmberg B Jansson R. Duodenal levodopa infusion monotherapy vs oral polypharmacy in advanced Parkinson disease. Neurology. (2005) 64:216–23. 10.1212/01.WNL.0000149637.70961.4C 15668416

[29] Gundert-Remy U Hildebrandt R Stiehl A Weber E Zürcher G Da Prada M. Intestinal absorption of levodopa in man. Eur J Clin Pharmacol. (1983) 25:69–72. 10.1007/BF00544017 6617727

[30] Camargo SMR Gotze O Singer D Verrey F Vuille-dit-Bille RN Ramadan T. The molecular mechanism of intestinal levodopa absorption and its possible implications for the treatment of Parkinson's disease. J Pharmacol Exp Ther. (2014) 351:114–23. 10.1124/jpet.114.216317 25073474

[31] Bröer S. Amino acid transport across mammalian intestinal and renal epithelia. Physiol Rev. (2008) 88:249–86. 10.1152/physrev.00018.2006 18195088

[32] Geier EG Chen EC Webb A Papp AC Yee SW Sadee W. Profiling solute carrier transporters in the human blood-brain barrier. Clin Pharmacol Ther. (2013) 94:636–9. 10.1038/clpt.2013.175 24013810 PMC3906042

[33] Wade LA Katzman R. Synthetic amino acids and the nature of L-DOPA transport at the blood-brain barrier. J Neurochem. (1975) 25:837–42. 10.1111/j.1471-4159.1975.tb04415.x 1206400

[34] Kageyama T Nakamura M Matsuo A Yamasaki Y Takakura Y Hashida M. The 4F2hc/LAT1 complex transports L-DOPA across the blood-brain barrier. Brain Res. (2000) 879:115–21. 10.1016/S0006-8993(00)02758-X 11011012

[35] Fraga S Serrão MP Soares-da-Silva P. L-type amino acid transporters in two intestinal epithelial cell lines function as exchangers with neutral amino acids. J Nutr. (2002) 132:733–8. 10.1093/jn/132.4.733 11925469

[36] Fraga S Sampaio-Maia B Serrão MP Soares-da-Silva P. Regulation of apical transporter of L-DOPA in human intestinal Caco-2 cells. Acta Physiol Scand. (2002) 175:103–11. 10.1046/j.1365-201X.2002.00974.x 12028130

[37] Fraga S Serrão MP Soares-da-Silva P. The L-3,4-dihydroxyphenylalanine transporter in human and rat epithelial intestinal cells is a type 2 hetero amino acid exchanger. Eur J Pharmacol. (2002) 441:127–35. 10.1016/S0014-2999(02)01416-4 12063083

[38] Fraga S Pinho MJ Soares-da-Silva P. Expression of LAT1 and LAT2 amino acid transporters in human and rat intestinal epithelial cells. Amino Acids. (2005) 29:229–33. 10.1007/s00726-005-0221-x 16027961

[39] Pinho MJ Serrão MP Gomes P Hopfer U Jose PA Soares-Da-Silva P. Over-expression of renal LAT1 and LAT2 and enhanced L-DOPA uptake in SHR immortalized renal proximal tubular cells. Kidney Int. (2004) 66:216–26. 10.1111/j.1523-1755.2004.00722.x 15200428

[40] Gomes P Soares-da-Silva P. Na+-independent transporters, LAT-2 and b0,+, exchange L-DOPA with neutral and basic amino acids in two clonal renal cell lines. J Membr Biol. (2002) 186:63–80. 10.1007/s00232-001-0136-8 11944084

[41] Bröer S. Xenopus laevis Oocytes. Methods Mol Biol. (2010) 637:295–310. 10.1007/978-1-60761-700-6_16 20419442

[42] Uchino H. Transport of amino acid-related compounds mediated by L-type Amino Acid Transporter 1 (LAT1): insights into the mechanisms of substrate recognition. Mol Pharmacol. (2002) 61:729–37. 10.1124/mol.61.4.729 11901210

[43] Quiñones H Collazo R Moe OW. The dopamine precursor l -dihydroxyphenylalanine is transported by the amino acid transporters rBAT and LAT2 in renal cortex. Am J Physiol Physiol. (2004) 287:F74–80. 10.1152/ajprenal.00237.2003 15180924

[44] Ishii H Sasaki Y Goshima Y Kanai Y Endou H Ayusawa D Involvement of rBAT in Na+-dependent and -independent transport of the neurotransmitter candidate L-DOPA in Xenopus laevis oocytes injected with rabbit small intestinal epithelium poly A+ RNA. Biochim Biophys Acta Biomembr. (2000) 1466:61–70. 10.1016/S0005-2736(00)00171-1 10825431

[45] Kim DK Kanai Y Chairoungdua A Matsuo H Cha SH Endou H. Expression cloning of a Na + -independent aromatic amino acid transporter with structural similarity to H + /Monocarboxylate transporters. J Biol Chem. (2001) 276:17221–8. 10.1074/jbc.M009462200 11278508

[46] Kim HT Edwards MJ Lakshmi Narsimhan R Bhatia KP. Hyperthyroidism exaggerating parkinsonian tremor: a clinical lesson. Parkinsonism Relat Disord. (2005) 11:331–2. 10.1016/j.parkreldis.2005.01.009 15970453

[47] Bianchine JR Messiha FS Hsu TH Peripheral aromatic L-amino acids decarboxylase inhibitor in parkinsonism. II. Effect on metabolism of L-2-^14^C-dopa. Clin Pharmacol Ther. (1972) 13:584–94. 10.1002/cpt1972134584 5042372

[48] Morgan JP. Metabolism of levodopa in patients with Parkinson's disease. Arch Neurol. (1971) 25:39. 10.1001/archneur.1971.00490010049007 5146410

[49] Sasahara K Nitanai T Habara T Kojima T Kawahara Y Morioka T. Dosage form design for improvement of bioavailability of levodopa IV: possible causes of low bioavailability of oral levodopa in dogs. J Pharm Sci. (1981) 70:730–3. 10.1002/jps.2600700705 7264915

[50] Iwamoto K Watanabe J Yamada M Atsumi F Matsushita T. Effect of age on gastrointestinal and hepatic first-pass effects of levodopa in rats. J Pharm Pharmacol. (1987) 39:421–5. 10.1111/j.2042-7158.1987.tb03413.x 2886598

[51] Robertson D Wood N Everest H Monks K Waller D Renwick A. The effect of age on the pharmacokinetics of levodopa administered alone and in the presence of carbidopa. Br J Clin Pharmacol. (1989) 28:61–9. 10.1111/j.1365-2125.1989.tb03506.x 2775615 PMC1379971

[52] Contin M Riva R Martinelli P Albani F Baruzzi A. Effect of age on the pharmacokinetics of oral levodopa in patients with Parkinson's disease. Eur J Clin Pharmacol. (1991) 41:463–6. 10.1007/BF00626370 1761075

[53] Nagayama H Ueda M Kumagai T Tsukamoto K Nishiyama Y Nishimura S. Influence of ageing on the pharmacokinetics of levodopa in elderly patients with Parkinson's disease. Park Relat Disord. (2011) 17:150–2. 10.1016/j.parkreldis.2010.11.002 21172738

[54] Daniel PM Moorhouse RS Pratt OE. Letter: do changes in blood levels of other aromatic aminoacids influence levodopa therapy? Lancet. (1976) 1:95. 10.1016/S0140-6736(76)90194-X 54620

[55] Nutt JG Woodward WR Hammerstad JP Carter JH Anderson JL. The on–off phenomenon in Parkinson's disease. N Engl J Med. (1984) 310:483–8. 10.1056/NEJM198402233100802 6694694

[56] Frankel JP Kempster PA Bovingdon M Webster R Lees AJ Stern GM. The effects of oral protein on the absorption of intraduodenal levodopa and motor performance. J Neurol Neurosurg Psychiatry. (1989) 52:1063–7. 10.1136/jnnp.52.9.1063 2795076 PMC1031741

[57] Nutt JG Woodward WR Carter JH Trotman TL. Influence of fluctuations of plasma large neutral amino acids with normal diets on the clinical response to levodopa. J Neurol Neurosurg Psychiatry. (1989) 52:481–7. 10.1136/jnnp.52.4.481 2738591 PMC1032296

[58] Leenders KL Poewe WH Palmer AJ Brenton DP Frackowiak RS Inhibition of L-[18F]fluorodopa uptake into human brain by amino acids demonstrated by positron emission tomography. Ann Neurol. (1986) 20:258–62. 10.1002/ana.410200212 3092728

[59] Lennernas H Nilsson D Aquilonius S Ahrenstedt O Knutson L Paalzow L. The effect of L-leucine on the absorption of levodopa, studied by regional jejunal perfusion in man. Br J Clin Pharmacol. (1993) 35:243–50. 10.1111/j.1365-2125.1993.tb05691.x 8471400 PMC1381569

[60] Cereda E Barichella M Pedrolli C Pezzoli G. Low-protein and protein-redistribution diets for Parkinson's disease patients with motor fluctuations: a systematic review. Mov Disord. (2010) 25:2021–34. 10.1002/mds.23226 20669318

[61] Guebila BM Thiele I. Model-based dietary optimization for late-stage, levodopa-treated, Parkinson's disease patients. Npj Syst Biol Appl. (2016) 2:16013. 10.1038/npjsba.2016.13 28725472 PMC5516849

[62] Goldin BR Peppercorn MA Goldman P. Contributions of host and intestinal microflora in the metabolism of L-dopa by the rat. J Pharmacol Exp Ther. (1973) 186:160–6. 4723308

[63] Sandler M Goodwin BL Ruthven CRJ Calne DB. Therapeutic implications in Parkinsonism of m-tyramine formation from L-dopa in man. Nature. (1971) 229:414–6. 10.1038/229414a0 4926994

[64] Quigley EMM Quera R. Small intestinal bacterial overgrowth: roles of antibiotics, prebiotics, and probiotics. Gastroenterology. (2006) 130:78–90. 10.1053/j.gastro.2005.11.046 16473077

[65] Gabrielli M Bonazzi P Scarpellini E Bendia E Lauritano EC Fasano A. Prevalence of small intestinal bacterial overgrowth in Parkinson's disease. Mov Disord. (2011) 26:889–92. 10.1002/mds.23566 21520278

[66] Lombardo L Foti M Ruggia O Chiecchio A. Increased incidence of small intestinal bacterial overgrowth during proton pump inhibitor therapy. Clin Gastroenterol Hepatol. (2010) 8:504–8. 10.1016/j.cgh.2009.12.022 20060064

[67] Freedberg DE Toussaint NC Chen SP Ratner AJ Whittier S Wang TC. Proton pump inhibitors alter specific taxa in the human gastrointestinal microbiome: a crossover trial. Gastroenterology. (2015) 149:883–5.e9. 10.1053/j.gastro.2015.06.043 26164495 PMC4584196

[68] Imhann F Bonder MJ Vich Vila A Fu J Mujagic Z Vork L. Proton pump inhibitors affect the gut microbiome. Gut. (2016) 65:740–8. 10.1136/gutjnl-2015-310376 26657899 PMC4853569

[69] Fried M Siegrist H Frei R Froehlich F Duroux P Thorens J. Duodenal bacterial overgrowth during treatment in outpatients with omeprazole. Gut. (1994) 35:23–6. 10.1136/gut.35.1.23 8307444 PMC1374626

[70] Steffen R. Rifaximin: a nonabsorbed antimicrobial as a new tool for treatment of travelers' diarrhea. J Travel Med. (2001) 8:S34–9. 10.1111/j.1708-8305.2001.tb00545.x 12186672

[71] Fasano A Bove F Gabrielli M Petracca M Zocco MA Ragazzoni E. The role of small intestinal bacterial overgrowth in Parkinson's disease. Mov Disord. (2013) 28:1241–9. 10.1002/mds.25522 23712625

[72] Pierantozzi M Pietroiusti A Galante A Sancesario G Lunardi G Fedele E. Helicobacter pylori-induced reduction of acute levodopa absorption in Parkinson's disease patients. Ann Neurol. (2001) 50:686–7. 10.1002/ana.1267 11706979

[73] Pierantozzi M Pietroiusti A Sancesario G Lunardi G Fedele E Giacomini P. Reduced L-dopa absorption and increased clinical fluctuations in Helicobacter pylori-infected Parkinson's disease patients. Neurol Sci. (2001) 22:89–91. 10.1007/s100720170061 11487216

[74] Narozanska E Białecka M Adamiak-Giera U Gawronska-Szklarz B Sołtan W Schinwelski M. Pharmacokinetics of levodopa in patients with parkinson disease and motor fluctuations depending on the presence of helicobacter pylori infection. Clin Neuropharmacol. (2014) 37:96–9. 10.1097/WNF.0000000000000037 24992088

[75] Lee WY Yoon WT Shin HY Jeon SH Rhee PL. Helicobacter pylori infection and motor fluctuations in patients with Parkinson's disease. Mov Disord. (2008) 23:1696–700. 10.1002/mds.22190 18649391

[76] Rahne KE Tagesson C Nyholm D. Motor fluctuations and Helicobacter pylori in Parkinson's disease. J Neurol. (2013) 260:2974–80. 10.1007/s00415-013-7089-6 24002418

[77] Lahner E Annibale B Delle Fave G. Systematic review: heliocobacter pylori infection and impaired drug absorption. Aliment Pharmacol Ther. (2009) 29:379–86. 10.1111/j.1365-2036.2008.03906.x 19053985

[78] Zar MA Ebong O Bateman DN. Effect of metoclopramide in guinea-pig ileum longitudinal muscle: evidence against dopamine-mediation. Gut. (1982) 23:66–70. 10.1136/gut.23.1.66 7056499 PMC1419580

[79] Görich R Weihrauch TR Kilbinger H The inhibition by dopamine of cholinergic transmission in the isolated guinea-pig ileum. Mediation through alpha-adrenoceptors Naunyn Schmiedebergs. Arch Pharmacol. (1982) 318:308–12. 10.1007/BF00501170 6281668

[80] Lucchelli A Boselli C Grana E Dopamine-induced relaxation of the guinea-pig isolated jejunum is not mediated through dopamine receptors. Pharmacol Res. (1990) 22:433–44. 10.1016/1043-6618(90)90750-8 1976247

[81] Kirschstein T Dammann F Klostermann J Rehberg M Tokay T Schubert R. Dopamine induces contraction in the proximal, but relaxation in the distal rat isolated small intestine. Neurosci Lett. (2009) 465:21–6. 10.1016/j.neulet.2009.08.080 19733212

[82] Zhang X Guo H Xu J Li Y Li L Zhang X. Dopamine receptor D1 mediates the inhibition of dopamine on the distal colonic motility. Transl Res. (2012) 159:407–14. 10.1016/j.trsl.2012.01.002 22500514

[83] Zizzo MG Mulè F Mastropaolo M Serio R. D1 receptors play a major role in the dopamine modulation of mouse ileum contractility. Pharmacol Res. (2010) 61:371–8. 10.1016/j.phrs.2010.01.015 20138148

[84] Auteri M Zizzo MG Amato A Serio R. Dopamine induces inhibitory effects on the circular muscle contractility of mouse distal colon via D1- and D2-like receptors. J Physiol Biochem. (2016) 73:395–404. 10.1007/s13105-017-0566-0 28600746

[85] Walker JK Gainetdinov RR Mangel AW Caron MG Shetzline MA. Mice lacking the dopamine transporter display altered regulation of distal colonic motility. Am J Physiol Gastrointest Liver Physiol. (2000) 279:G311–8. 10.1152/ajpgi.2000.279.2.G311 10915639

[86] Fioramonti J Fargeas MJ Honde C Bueno L. Effects of central and peripheral administration of dopamine on pattern of intestinal motility in dogs. Dig Dis Sci. (1984) 29:1023–7. 10.1007/BF01311254 6489081

[87] Bueno L Fargeas MJ Fioramonti J Honde C. Effects of dopamine and bromocriptine on colonic motility in dog. Br J Pharmacol. (1984) 82:35–42. 10.1111/j.1476-5381.1984.tb16439.x 6145468 PMC1987238

[88] Marzio L Neri M Di Giammarco AM Cuccurullo F Lanfranchi GA. Dopamine-induced migrating myoelectrical complex-like activity in human duodenum. Dig Dis Sci. (1986) 31:349–54. 10.1007/BF01311668 3956330

[89] Marzio L Neri M Pieramico O Donne MD Peeters TL Cuccurullo F. Dopamine interrupts gastrointestinal fed motility pattern in humans. Dig Dis Sci. (1990) 35:327–32. 10.1007/BF01537410 1968372

[90] Levein NG Thörn SE Wattwil M. Dopamine delays gastric emptying and prolongs orocaecal transit time in volunteers. Eur J Anaesthesiol. (1999) 16:246–50. 10.1097/00003643-199904000-00006 10234494

[91] Dive A Foret F Jamart J Bulpa P Installé E. Effect of dopamine on gastrointestinal motility during critical illness. Intensive Care Med. (2000) 26:901–7. 10.1007/s001340051279 10990104

[92] Li ZS Schmauss C Cuenca A Ratcliffe E Gershon MD. Physiological modulation of intestinal motility by enteric dopaminergic neurons and the D2 receptor: analysis of dopamine receptor expression, location, development, and function in wild-type and knock-out mice. J Neurosci. (2006) 26:2798–807. 10.1523/JNEUROSCI.4720-05.2006 16525059 PMC6675162

